# The effect of oral magnesium supplementation on glycemic control and metabolic parameters in type 2 diabetes mellitus: a double-blind randomized controlled trial

**DOI:** 10.3389/fendo.2026.1883483

**Published:** 2026-07-16

**Authors:** Juhaina Salim Al-Maqbali, Abdullah M. Al Alawi, Alaa Osman, Ahmed Al-Farqani, Suneel Kumar, Abdullah Al Futisi, Ali Al-Mamari, Ibrahim Al-Zakwani, Mohammed Al Za’abi

**Affiliations:** 1 Department of Pharmacology and Clinical Pharmacy, College of Medicine and Health Science, Sultan Qaboos University, Muscat, Oman; 2 Department of Pharmacy, Sultan Qaboos University Hospital, University Medical City, Muscat, Oman; 3 Department of Medicine, Sultan Qaboos University Hospital, University Medical City, Muscat, Oman; 4 Department of Medicine, Sultan Qaboos University, Muscat, Oman

**Keywords:** glycated hemoglobin, hypomagnesemia, intention-to-treat analysis, magnesium oxide, per-protocol analysis

## Abstract

**Background:**

This randomized controlled trial (RCT) assessed the effect of oral magnesium (Mg) supplementation on glycated hemoglobin (HbA1c) and metabolic biomarkers in adults with Type 2 Diabetes Mellitus (T2DM).

**Methods:**

This was a prospective, randomized, double-blind, placebo-controlled, parallel-group clinical trial with intention-to-treat (ITT) and per-protocol (PP) analyses. Adults (≥18 years) with T2DM were randomized 1:1 to receive magnesium oxide (500 mg; 302 mg elemental) or placebo. Eligibility criteria included a glycated hemoglobin (HbA1c) level ≥7.0% and a creatinine clearance >30 mL/min. Simple randomization was performed using a sealed-envelope method. Biochemical and clinical parameters were assessed at baseline and at three follow-up visits over 12 months.

**Results:**

A total of 247 participants were included in the ITT analysis (Mg-oxide, n=118; placebo, n=129). Median age was 58 (50–65) years, and median diabetes duration was 16 (10–22) years. Hypomagnesemia was present in 7.3% by ionized magnesium (iMg) and 8.1% by total magnesium (tMg). Mg-oxide supplementation significantly reduced fasting blood glucose in a subgroup of the cohort (FBG; *p* = 0.039) and resulted in a modest, non-significant reduction in HbA1c (−0.30% vs. −0.05%; *p* = 0.145), with a higher proportion achieving goal attainment/controlled HbA1c (<7%) compared with placebo (14.0% vs. 6.0%; *p* = 0.043). HbA1c reductions were more pronounced among participants with baseline hypomagnesemia (−0.60% vs. +0.25%; *p* = 0.074), or diabetes duration ≤15 years (−0.40% vs. 0.00%; *p* = 0.085). No differences were observed in other metabolic parameters, clinical outcomes or adverse drug reactions between groups.

**Conclusions:**

Although median HbA1c reduction did not reach statistical significance, Mg-oxide supplementation improved Mg status and showed favorable trends in glycemic control without compromising safety. These findings support Mg supplementation as a safe adjunct to long-term T2DM management, particularly among selected subgroups, given the study’s limitations.

**Clinical Ttrial Registration:**

https://clinicaltrials.gov/, identifier NCTO5774015.

## 1 Introduction

Management of diabetes aims to achieve durable glycemic control and prevent microvascular and macrovascular complications. The American Diabetes Association emphasizes a patient-centered approach combining lifestyle modification, pharmacologic therapy, and comprehensive risk-factor control (1, 2). In type 2 diabetes mellitus (T2DM), treatment typically begins with oral hypoglycemic agents (OHAs) targeting multiple regulatory pathways involving insulin, glucagon, amylin, glucagon-like peptide-1 (GLP-1), and glucose-dependent insulinotropic polypeptide (GIP) (3). These include insulin secretagogues, metformin, insulin sensitizers, alpha-glucosidase inhibitors, incretin-based therapies, amylin analogues, and sodium-glucose cotransporter 2 (SGLT2) inhibitors (3). Emerging therapies, such as dual GIP/GLP-1 receptor agonists, have demonstrated substantial metabolic benefits (4). Treatment is individualized according to glycemic status, glycated hemoglobin (HbA1c), and fasting blood glucose (FBG), while adjunctive strategies, including vitamin D and magnesium (Mg) supplementation, may offer modest additional benefits (5).

Mg is a cofactor for over 300 enzymatic reactions and has a fundamental role in cellular physiology (6). Approximately 1% of total body Mg is present in the circulation, of which 65–70% exists as biologically active ionized Mg (iMg). Mg homeostasis is tightly regulated by intestinal absorption and renal excretion, maintaining plasma concentrations within a narrow range of 0.7–1.0 mmol/L (7). Disturbances in Mg balance are clinically important; hypomagnesemia is common in hospitalized and diabetic populations, whereas hypermagnesemia occurs primarily in renal impairment or excessive supplementation (8). Chronic Mg deficiency disrupts energy metabolism and promotes hyperinsulinemia and insulin resistance, thereby increasing the risk of T2DM. A large meta-analysis demonstrated that each additional 100 mg/day of dietary Mg intake is associated with an approximately 15% reduction in T2DM risk (9). In individuals with diabetes, Mg is essential for insulin signaling, glucose transport, and pancreatic β-cell function, and deficiency is linked to poor glycemic control, dyslipidemia, and increased cardiometabolic risk (10, 11). Both total Mg (tMg) and iMg predict clinical outcomes, although iMg appears to show stronger and more consistent associations (12–14).

Randomized controlled trials (RCTs) of Mg supplementation in T2DM have yielded mixed but informative results. An early double-masked RCT from Mexico (2003) reported that T2DM patients on OHAs with low tMg (≤0.74 mmol/L) experienced significant increases in tMg and concurrent improvement in FBG and HbA1c after 16 weeks of MgCl₂ (15). Subsequent trials by Razzaghi R et al., 2018 demonstrated that 12 weeks of Mg-oxide (250 mg/day) significantly reduced FBG and HbA1c (16). Conversely, a 2019 double-blind RCT in patients with T2DM, diabetic nephropathy, and hypomagnesemia reported no significant changes in tMg or HbA1c, suggesting heterogeneity related to renal dysfunction and Mg handling (17). A meta-analysis of RCTs conducted between 1989 and 2022 (n=1694) found no overall significant reduction in HbA1c among T2DM populations, although consistent benefits were observed in those with baseline hypomagnesemia (5, 18). A short-term Serbian RCT further showed that only Mg-oxide increased both iMg and tMg, whereas Mg citrate and Mg carbonate resulted in discordant Mg fraction responses (19). Beyond glycemic outcomes, Mg supplementation has demonstrated favorable effects on blood pressure (BP) and lipid profiles (5, 18).

To the best of our knowledge, no RCT has evaluated the long-term (12-month) effect of Mg-oxide supplementation guided by iMg concentrations in T2DM. Existing iMg guided trials were conducted in healthy volunteers and were short-term (a few days to 10 weeks) (19, 20), whereas RCTs of Mg-oxide in T2DM were guided by tMg and usually limited to 12 weeks (18). Given Mg-oxide solubility is strongly pH dependent (21), reliance on tMg alone may miss functional deficiency, especially when iMg or the iMg/tMg ratio is reduced (13). Accordingly, this RCT was designed to evaluate the effect of Mg-oxide versus placebo in patients with T2DM (with or without hypomagnesemia) on glycemic control (FBG, HbA1c, and total insulin dose), prognosis (lipid profile, BP, body mass index [BMI] and serum creatinine), and clinical outcomes (emergency department [ED] visits, hospital admissions, and length of hospital stay [LOS]), using both serum iMg and tMg as guiding parameters.

## 2 Materials and methods

### 2.1 Trial design, setting and population

This was a prospective, randomized, double-blind, placebo-controlled, parallel-group clinical trial conducted in adult patients (≥18 years) with T2DM attending the endocrinology clinic at Sultan Qaboos University Hospital (SQUH). Participants were randomly assigned in a 1:1 ratio to receive Mg-oxide supplements or identical placebo tablets for 12 months.

### 2.2 Eligibility criteria

Adults (≥18 years) with T2DM treated with OHA with or without insulin regimen, and with at least one HbA1c result within the last 6 months were included.

Patients were excluded if they had Type 1 diabetes; Gestational diabetes mellitus (GDM); or maturity-onset diabetes of the young; HbA1c <7%; were fully dependent or unable to communicate; with creatinine clearances (CrCl) < 30 mL/min; active solid or hematological malignancies; cognitive disorders or psychiatric illness; post-transplanted; pregnancy; use of Mg supplementation within the past 3 months; or use of contraindicated medications to Mg like baloxavir marboxil, calcium/sodium polystyrene sulfate, raltegravir, or unithiol.

### 2.3 Outcomes measures

#### 2.3.1 Primary outcomes (disease control)

The primary outcome was changed in the HbA1c at 12-month of supplementation between the Mg-oxide and Placebo groups.

#### 2.3.2 Secondary outcomes (prognosis, clinical outcomes, safety)

Secondary outcomes included changes in FBG, total daily insulin dose, BP, lipid profile, serum creatinine, BMI, number of ED visits, hospital admissions, and LOS over 12 months. Safety outcomes included the incidence of adverse drug reactions (ADRs).

### 2.4 Sample size

The sample size was calculated based on the primary outcome, HbA1c reduction. Previous research by Martha et al., 2003 (15), ELDerawi et al., 2018 (22), and Razzaghi R et al., 2018 (16) reported relative HbA1c reductions of -1.7%, -0.36%, and -0.7%, respectively, after 12 weeks of supplementation with Mg. We predicted a 1% decrease in HbA1c from 9% to 8% with a standard deviation of 3% and 2%, a power of 80% and an alpha level of 5%, 103 patients per group (total n= 206) were required. To account for any anticipated loss to follow-up, the study’s sample size increased by approximately 20% to a total of 247 patients.

### 2.5 Study protocol

#### 2.5.1 Randomization, and blinding

Simple randomization was conducted using a sealed-envelope method. Tables and labels were generated using STATA software version 16.1 (StataCorp, College Station, TX, USA). Labels remained concealed until after the participant’s written consent and contact details had been obtained. Participants, healthcare providers, outcome assessors, and data analysts were blinded to group assignment.

#### 2.5.2 Recruitment, intervention and comparator

Eligible patients were identified from the Trak-Care^©^ system at SQUH and approached during clinic visits. After obtaining consent, participants were randomized to receive placebo or 500 mg Mg-oxide (302 mg elemental). Tablets were taken once nightly with food, ≥2 hours apart from other medications, for 12 months. Participants maintained usual diet, avoided Mg supplements, and reported ADRs (18–20, 23, 24).

#### 2.5.3 Follow-up visits and procedures

Post 1-month: Telephone follow-up assessed self-reported adherence using a single-item measure. Post 3-, 6-, or 9-month: Laboratory tests were repeated; ADRs, ED visits, and admissions recorded; adherence assessed using the Morisky Medication Adherence Scale – 8 items (MMAS-8), ^©^ 2007 Donald E. Morisky provided the MMAS-8 Scale permission for use (Certification number: 1350) (25, 26); remaining bottles dispensed. Post 12-month: Final assessment, labs, optional survey, and discontinuation.

### 2.6 Mg-oxide tablets and placebo tablets

Mg-oxide and placebo tablets were identical in appearance, smell, and labeling (500 mg, 302 mgelemental) and had a 24-month shelf life from the manufacturer. Tablets were manufactured and packaged by Dietary Supplement Contract Manufacturing Services, Istanbul, Türkiye (www.mirfarma.com.tr). Details of the tablet ingredients are provided in **Supplementary Table 1**.

Although Mg-oxide has lower bioavailability than some organic salts, it was selected because of its high elemental Mg content, availability, cost-effectiveness, and widespread clinical use. Previous RCTs in T2DM have demonstrated favorable glycemic effects, particularly in patients with hypomagnesemia. Additionally, RCTs showed that Mg-oxide consistently increased both iMg and tMg, supporting its suitability for our iMg-guided study.

### 2.7 Definitions

Hypomagnesemia measured by tMg was defined as serum tMg concentration <0.7 mmol/lL while hypermagnesemia was defined as serum tMg concentration >1.0 mmol/L (12). Hypomagnesemia was defined as serum iMg concentration <0.47 mmol/L, and hypermagnesemia as serum iMg concentration >0.68 mmol/L, based on the recent study that established the Omani reference range (12). Goal attainment was defined as achieving a controlled HbA1c (<7%) post-supplementation (27).

### 2.8 Laboratory measurements

Investigations included FBG; mmol/L, HbA1c; % (blood samples for HbA1c measurement were collected in ethylenediaminetetraacetic acid (EDTA) tubes, serum creatinine; mmol/L, serum calcium and ionized calcium (iCa); mmol/L, lipid profile (low-density lipoprotein [LDL], high-density lipoprotein [HDL], triglycerides [TG], and total cholesterol); mmol/L, serum tMg and iMg, mmol/L, systolic BP (SBP) and diastolic BP (DBP); mmHg.

Blood samples for iMg and iCa were collected by a trained nurse and analyzed using Stat Profile Prime Plus^®^ (Nova Biomedical, Waltham, MA, USA). The syringes used were heparinized with 60 IU heparin (self-filling sampler by Radiometer^®^, 2700 Bronshoj, Denmark). The device employs a direct ion-selective electrode (ISE) method with ionophore-based detection. This validated potentiometric technique (28) enables rapid, accurate measurements using small volumes. Measuring ranges were 0.1–1.5 mmol/L for iMg and 0.1–2.7 mmol/L for iCa.

### 2.9 Data collection and handling missingness

Collected variables included: demographic characteristics (age, sex, BMI), duration of T2DM, comorbidities (hypertension, heart failure, cardiovascular disease, chronic kidney disease), smoking and alcohol history, baseline microvascular complications, and details of the diabetes regimen. Baseline and follow-up clinical and laboratory parameters (FBG, HbA1c, serum creatinine, calcium, iMg, tMg, lipid profile, BP), ED visits, hospital admissions (including LOS), ADRs, adherence, and reasons for withdrawal were recorded.

Missing data for participants who withdrew were handled in accordance with U.S. Food and Drug Administration (FDA) guidance using last-observation-carried-forward (LOCF). LOCF prior to dropout was carried forward for all subsequent follow-up time points for participants who declined follow-up after stopping the intervention (29).

### 2.10 Withdraw criteria, and population analysis

Participants were withdrawn if they developed dysmagnesemia requiring treatment, withdrew voluntarily before 12 months, were lost to follow-up, or died; all were included in *Intention-to-Treat* (ITT). Participants completing 12 months without major protocol deviations, regardless of adherence, were included in the *Per-Protocol* (PP) analysis.

Missing data due to withdraw were handled in accordance with U.S. Food and Drug Administration (FDA) guidance using last-observation-carried-forward.

### 2.11 Criteria for terminating the study

Prespecified stopping guidelines allowed early termination of the trial if any intervention was associated with an unacceptable risk to participants, including serious adverse events or unexpected harm that outweighed potential benefit. Decisions followed the Consolidated Standards of Reporting Trials (CONSORT) principles.

### 2.12 Statistical analysis

Categorical variables were summarized as frequencies and percentages. Continuous variables were described using the mean and standard deviation for normally distributed data, or the median and interquartile range for non-normally distributed data.

Both ITT and PP analyses were performed (29) to assess primary and secondary outcomes. Between-group comparisons for continuous variables used Student’s t-test or Mann–Whitney U test, as appropriate, while categorical variables were analyzed using Pearson’s χ^2^ or Fisher’s exact test. Within-group changes were assessed using paired t-test or Wilcoxon signed-rank test, and McNemar’s χ^2^ test for Mg status. Agreement between iMg and tMg was evaluated using Spearman’s correlation and Lin’s concordance. Two-tailed level of significance was set at *p* < 0.05. Statistical analyses were conducted using Stata version 16.1 (StataCorp, College Station, TX, USA).

All statistical analyses were reviewed and verified by an independent biostatistician.

## 3 Results

### 3.1 Participant screening, recruitment, and follow-up

**Figure 1:**
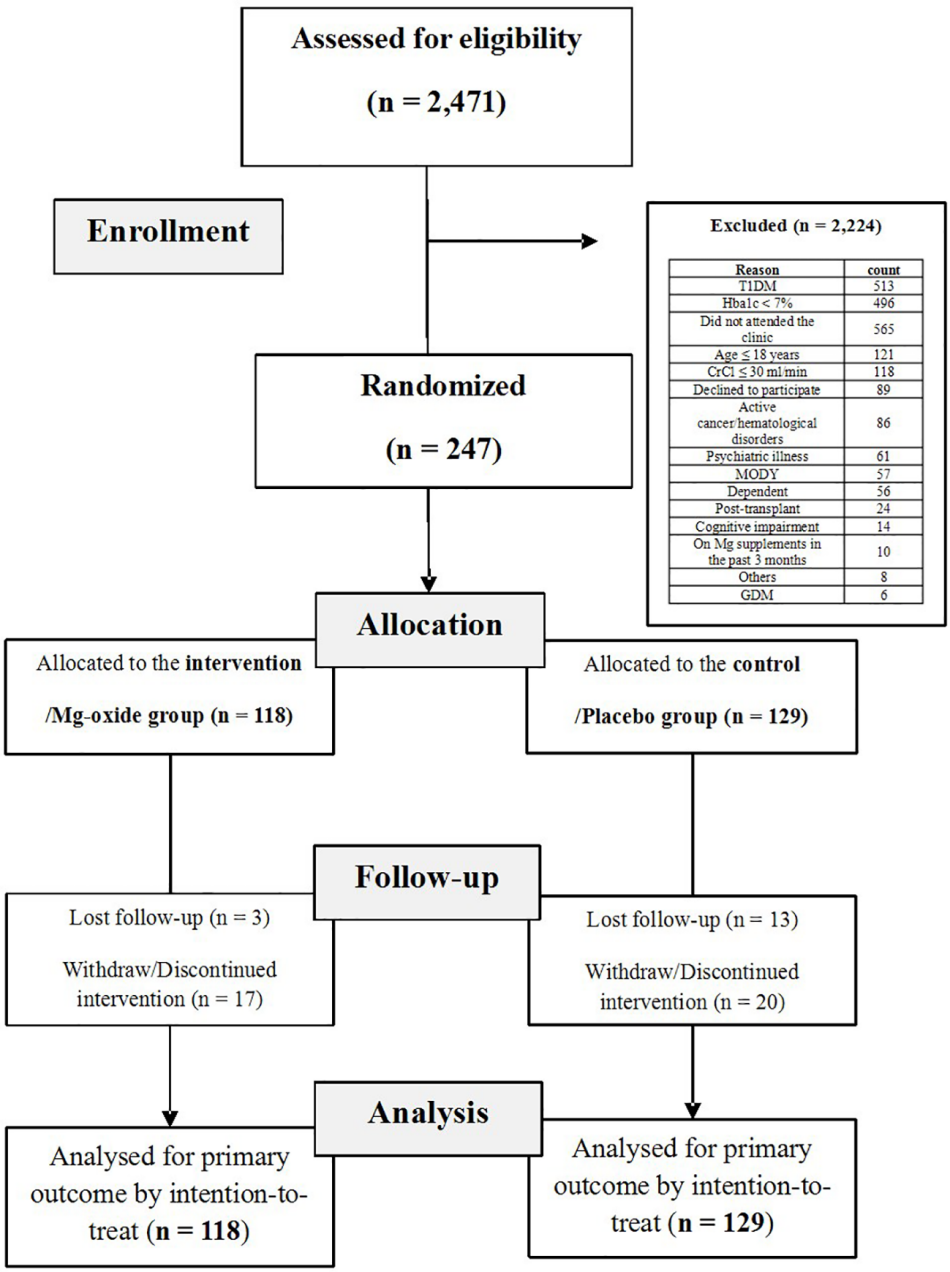
The CONSORT flow diagram for the study inclusion, exclusion and analysis.

Between September 25, 2023, and September 30, 2024, 36 recruitment clinics were conducted, followed by 50 follow-up clinics between December 27, 2023, and September 15, 2025. As reported in the CONSORT figure, of the 2,471 patients assessed for eligibility, 2,224 subjects were excluded. The most common exclusions were T1DM (n = 513) or HbA1c <7% (n = 496). Only 89 patients declined to participate, and 247 were randomized and included in the ITT analysis (n=118 Mg-oxide-group *vs*. n=129 placebo-group) (**Figure 1**).

While 194 participants (79%) completed the 12-month follow-up without major protocol deviation and were included in the PP analysis. Among the remaining, 37 discontinued the intervention after the first or second follow-up, 15 were lost to follow-up, and one participant died nine months after recruitment (**Figure 1**).

### 3.2 Baseline clinical and laboratory characteristics

Among the 247 randomized participants, females accounted for 53.0% (n = 131). The median age was 58 years (IQR 50–65), median BMI was 30.1 kg/m^2^ (IQR 26.7–33.8), and the median diabetic duration was 16 years (IQR 10–22) years. The median HbA1c was 8.8 (IQR 8.0–10.3)%, and 176 participants (71.3%) had hypertension. The mean baseline iMg and tMg were 0.60 ± 0.09 mmol/L and 0.82 ± 0.09 mmol/L, respectively. Clinical and laboratory characteristics were generally well balanced between groups, except for metformin use, which was significantly higher in the Mg-oxide group (96.61% *vs.* 89.92%; *p* = 0.038) (**Table 1**).

**Table 1:** Clinical characteristics, laboratory profile and medications use of the study participants (n = 247).

Characteristic *n (%) unless specified otherwise*	Total 247 (100)	Placebo group 129 (52.23)	Mg-oxide group 118 (47.77)	*p*-value
Withdraw and loss of follow-up	53 (21.46)	33 (25.58)	20 (16.95)	0.099
Females	131 (53.04)	72 (55.81)	59 (50.00)	0.360
Age, median (IQR); years	58 (50–65)	58 (50–64)	59 (50–65)	0.696
BMI, median (IQR); kg/m^2^	30.1 (26.7–33.8)	30.3 (26.1–34.0)	30.1 (27.1–33.8)	0.908
DM history and complication				
Duration of DM, median (IQR); years	16 (10–22)	16 (10–21)	18 (10–23)	0.597
FBG, median (IQR); mmol/l	8.6 (7.0–10.7)	9.2 (7.0–12.0)	8.3 (7.15–9.7)	0.067*
HbA1c, median (IQR); %	8.8 (8.0–10.3)	8.9 (8.2–10.3)	8.7 (8.0–10.2)	0.370
Diabetic nephropathy	76 (30.77)	40 (31.01)	36 (30.51)	0.932
Diabetic retinopathy	68 (27.53)	37 (28.68)	31 (26.27)	0.672
Diabetic neuropathy and on Vit B complex	34 (13.77)	20 (15.50)	14 (11.86)	0.407
Other medical history				
Smoking	7 (2.83)	3 (2.33)	4 (3.39)	0.615
Hypertension	176 (71.26)	87 (67.44)	89 (75.42)	0.166
Systolic blood pressure, median (IQR); mmHg	135 (123–148)	134 (123–148)	135 (123–149)	0.959
Diastolic blood pressure, median (IQR); mmHg	72 (65–82)	73 (65–84)	72 (66–81)	0.684
Chronic Kidney Disease (CKD)	27 (10.93)	12 (9.30)	15 (12.71)	0.391
Thyroid disease	40 (16.19)	16 (12.40)	24 (20.34)	0.091
Heart failure	8 (3.24)	5 (3.88)	3 (2.54)	0.554
Ischemic heart disease	13 (5.26)	8 (6.20)	5 (4.24)	0.490
Dyslipidemia	165 (66.80)	89 (68.99)	76 (64.41)	0.445
Laboratory data				
TG, median (IQR); mmol/l	1.5 (1.1–2.1)	1.4 (1.1–2.1)	1.5 (1.2–2.2)	0.223
LDL, median (IQR); mmol/l	2.2 (1.6–3.0)	2.4 (1.7–3.0)	2.2 (1.6–3.0)	0.280
HDL, median (IQR); mmol/l	1.20 (1.02–1.50)	1.24 (1.04–1.54)	1.16 (0.97–1.43)	0.167
Lung disease	18 (7.29)	9 (6.98)	9 (7.63)	0.844
Plasma albumin, median (IQR); mmol/l	44 (42–46)	44 (42–46)	45 (43–46)	0.296
Albumin/Creatinine ratio, median (IQR); mg/mmol	2.31 (2.26–2.38)	2.31 (2.25–2.36)	2.33 (2.26–2.39)	0.328
Serum adjusted calcium, median (IQR); mmol/l	1.18 (1.06–1.32)	1.17 (1.04–1.32)	1.21 (1.10–1.32)	0.532
Serum phosphate, median (IQR); mmol/l	1.8 (0.5–9.5)	1.6 (0.7–11.2)	1.8 (0.5–7.9)	0.403
Serum sodium, median (IQR); mmol/l	139 (137–141)	139 (137–141)	139 (137–140)	0.981
Serum potassium, median (IQR); mmol/l	4.5 (4.3–4.8)	4.5 (4.3–4.7)	4.5 (4.3–4.8)	0.889
Serum iMg, mean ± SD; mmol/l	0.60 (0.09)	0.59 (0.09)	0.61 (0.10)	0.143
Serum tMg, mean ± SD; mmol/l	0.82 (0.09)	0.82 (0.09)	0.82 (0.08)	0.958
Serum creatinine, median (IQR); mmol/l	70 (56–85)	71 (54–82)	69 (57–90)	0.546
eGFR, median (IQR); mL/min/1.73 m^2^	90 (74–90)	90 (79–90)	90 (74–90)	0.377
Diabetes medications				
Insulin	152 (61.54)	81 (62.79)	71 (60.17)	0.672
Insulin dose, IQR; unit	60 (32–85)	60 (30–86)	60 (36–84)	0.933
Metformin	230 (93.12)	116 (89.92)	114 (96.61)	0.038
Metformin dose, IQR; mg	2000 (2000–2000)	2000 (2000–2000)	2000 (2000–2000)	0.289
Sulfonylureas (Gliclazide)	140 (56.68)	72 (55.81)	68 (57.63)	0.774
Sulfonylurea dose, IQR; mg	120 (60–120)	120 (60–120)	120 (60–120)	0.325
SGLT2 inhibitor (Dapagliflozin)	195 (78.95)	104 (80.62)	91 (77.12)	0.500
SGLT2 dose, IQR; mg	10 (10–10)	10 (10–10)	10 (10–10)	1.000
GLP-1A (Liraglutide)	36 (14.57)	16 (12.40)	20 (16.95)	0.312
GLP-1A dose, IQR; mg	1.8 (1.8–1.8)	1.8 (1.8–1.8)	1.8 (1.8–1.8)	0.873
Other medications				
Loop diuretics (frusemide)	11 (4.45)	9 (6.98)	2 (1.69)	0.044
Thiazides (hydrochlorothiazide)	85 (34.41)	41 (31.78)	44 (37.29)	0.363
Proton pump inhibitors (esomeprazole)	89 (36.03)	48 (37.21)	41 (34.75)	0.687

IQR, interquartile range; SD, standard deviation; BMI, body mass index; DM, diabetes mellitus; FBG, fasting blood glucose; HbA1c, glycated hemoglobin; SBP, systolic blood pressure; DBP, diastolic blood pressure; CKD, chronic kidney disease; TG, triglycerides; LDL, low-density lipoprotein; HDL, high-density lipoprotein; Ca, calcium; iMg, ionized magnesium; tMg, total magnesium; eGFR, estimated glomerular filtration rate; SGLT2, sodium–glucose cotransporter-2; GLP-1A, glucagon-like peptide-1 agonist; *Total observation is this group is n = 147 only (Placebo group=71, Mg-oxide group=76). The Wilcoxon-Mann-Whitney test was used to ascertain the association between the continuous variables (with abnormal distribution) and the groups, while the two-sample t-test was used when the distribution was normal. The associations between categorical variables and groups were determined by the Chi-square test or Fisher’s exact test when cells fall below 5.

### 3.3 Medication adherence assessment

At 3-months, the median MMAS-8 score was 7 (IQR 6–8). A significantly greater proportion of participants in the Mg-oxide group achieved high adherence compared with placebo (49.15% *vs.* 35.66%; *p* = 0.032), whereas, low adherence was significantly less frequent (14.41% *vs.* 26.36%; *p* = 0.020).

### 3.4 Prevalence and changes in Mg status

At baseline, the prevalence of hypomagnesemia based on iMg was 7.3% (95% confidence interval[CI]: 4.6%, 11.3%), while tMg-defined hypomagnesemia was observed in 8.1% (95% CI: 5.3%, 12.2%).Over 12 months, the distribution of Mg status shifted modestly. As shown in (**Supplementary Table 2**), using iMg, the proportion of participants with hypomagnesemia declined slightly from 7.3% at baseline to 4.1% at 12 months, a statistically significant within-group change (McNemar’s χ^2^ = 6.40, *p* = 0.011).

### 3.5 Agreement and correlation between Mg measures and glycemic control

Both iMg and tMg showed weak negative correlations with HbA1c at baseline and follow-up. Theinverse correlation between iMg and baseline HbA1c was statistically significant but weak(Spearman’s r = –0.1983; *p* < 0.001), whereas this correlation was no longer significant at the 12-month follow-up (Spearman’s *r* = –0.0919; *p* = 0.15). In contrast, tMg showed a nonsignificant correlation with HbA1c at either time point (*p* > 0.05) (**Supplementary Table 3**).

### 3.6 Biochemical and clinical outcomes

In the ITT cohort (n = 247), Mg-oxide produced a modest, non-significant improvement in glycemic indices. Median HbA1c change was −0.25% [IQR −1.10 – 0.40] in the Mg-oxide group *vs.* 0.00% [−0.80 – 0.50] in the placebo group (*p* = 0.181) (**Table 2**). A higher proportion of participants in the Mg-oxide group achieved the goal attainment of HbA1c <7%; however, this difference was only marginally significant (13.6% *vs*. 6.2%; *p* = 0.051). FBG decreased significantly in the Mg-oxide group compared with the placebo group (-0.4 *vs.* 0 mmol/L; *p* = 0.035).

**Table 2:** Biochemical, clinical, and safety outcomes-supplementations based on intension to treat analysis (n = 247).

Characteristic *n (%) unless specified otherwise*	Total 247 (100)	Placebo group 129 (52.23)	Mg-oxide group 118 (47.77)	*p*-value
Mg concentration				
Δ iMg, IQR; mmol/l	−0.01 (−0.01 – 0.09)	0 (0 – 0.10)	−0.01 (0.01 – 0.07)	0.355
Δ tMg, IQR; mmol/l	0 (−0.03 – 0.05)	−0.04 (0 – 0.05)	−0.02 (0 – 0.05)	0.686
Diabetic control				
Δ HbA1c, IQR; %	0.2 (−1.0 – 0.5)	0.0 (−0.8 – 0.5)	−0.25 (−1.1 – 0.4)	0.181
Controlled HbA1c (<7%)	24 (9.7%)	8 (6.2%)	16 (13.6%)	0.051
Δ FBG, IQR; mmol/l	0 (−1.6 – 0.8)	0 (−0.6 – 1.25)	−0.4 (−2.2 – 0.5)	0.035*
Diabetic prognosis				
Δ SBP, (± SD); mmHg	−1.36 (± 18.38)	−0.78 (± 18.65)	−1.99 (± 18.15)	0.605
Δ DBP, (± SD); mmHg	−1.44 (± 12.69)	−1.58 (± 12.74)	−1.28 (± 12.69)	0.852
Δ BMI, IQR; kg/m2	−0.05 (± 1.96)	−0.21 (± 1.67)	0.13 (± 2.22)	0.175
Δ TG, IQR; mmol/l	0 (−0.30 – 0.30)	0 (−0.20 – 0.40)	0 (−0.40 – 0.27)	0.136
Δ LDL, IQR; mmol/l	0 (−0.40 – 0.30)	0 (−0.40 – 0.30)	0 (−0.30 – 0.30)	0.343
Δ HDL, IQR; mmol/l	−0.01 (−0.12 – 0.06)	−0.03 (−0.13 – 0.05)	0 (−0.09 – 0.08)	0.062
Δ Creatinine, IQR; mmol/l	0 (−4 – 5)	0 (−4 – 4)	0 (−4 – 6)	0.593
Δ Serum eGFR, IQR; mL/min/1.73 m^2^	0 (−4 – 0)	0 (−2 – 0)	0 (−4 – 0)	0.331
Δ Albumin/Creatinine ratio, IQR; mg/mmol	0 (0 – 0.10)	0 (0 – 0)	0 (0 – 0.20)	0.549**
Δ Insulin dose, IQR; mg	0 (0 – 12)	0 (0 – 2)	0 (0 – 20)	0.126
ADRs associated with Mg-oxide or placebo supplements				
Total Number of all ADRs	90 (36.18)	48 (37.21)	42 (35.59)	0.792
Diarrhea	39 (15.79)	21 (16.28)	18 (15.25)	0.863
Clinical outcomes				
Emergency visits	33 (17.0)	17 (17.7)	16 (16.3)	0.798
Hospital admissions	17 (8.8)	9 (9.4)	8 (8.2)	0.765
Length of hospital stay; (± SD)	0.42 (± 2.02)	0.31 (± 1.36)	0.54 (± 2.56)	0.369

iMg, ionized magnesium; tMg, total magnesium; HbA1c, glycated hemoglobin; FBG, fasting blood glucose; SBP, systolic blood pressure; DBP, diastolic blood pressure; BMI, body mass index; TG, triglycerides; LDL, low-density lipoprotein; HDL, high-density lipoprotein; eGFR, estimated glomerular filtration rate; SD, standard deviation; IQR, interquartile range; ADRs, adverse drug reactions.

ADRs include (abdominal pain, cramps, sleepiness, lethargy, dyspepsia, chest pain, constipation, pimples±, allergic itchiness, and nausea).

*Total observation is this group is n = 67 only.

**Total observation is this group is n =82 only.

The Wilcoxon-Mann-Whitney test was used to determine the association between the continuous variables (with abnormal distributions) and the groups. In contrast, the two-sample t-test was used when the distributions were normal. The associations between categorical variables and groups were determined by the Chi-square test or Fisher’s exact test when cells fall below 5.

No significant between-group differences were observed in SBP, DBP, lipid profile, BMI, renal indices (creatinine, estimated glomerular filtration rate [eGFR], albumin-to-creatinine ratio), or insulin dose changes (all *p* > 0.05). (**Table 2**).

After excluding participants who were not receiving metformin at baseline, the analysis was restricted to those using metformin (n = 230), most metabolic, cardiovascular, renal, and safety outcomes remained similar between groups. Median HbA1c reduction favored Mg-group (−0.30% *vs*. −0.05%; *p* = 0.145), and a significantly higher proportion of participants achieved controlled HbA1c (<7%) with Mg-oxide compared with placebo (14.0% *vs*. 6.0%; *p* = 0.043). Mg-oxide also produced a greater median reduction in FBG (−0.40 *vs*. 0.00 mmol/L; *p* = 0.039).

PP analysis (n =194; 65 in Mg-oxide *vs.* 61placebo) supported the ITT findings, with a larger reduction in FBG in the Mg-oxide group (−0.75 mmol/L [−2.4 to 0.5] *vs*. 0.8 mmol/L [−0.5 to 2.1]; *p* = 0.006). Median HbA1c changes were −0.3% [−1.1 to 0.3] % *vs*. −0.2% [−0.85 to 0.65] %, (*p* = 0.19). Other metabolic markers showed no significant between-group differences.

### 3.7 Safety outcomes

Overall, 90 participants (36.4%) reported at least one ADR associated with either Mg-oxide or placebo, with no significant difference between groups (*p* = 0.792). Diarrhea was the most common ADR, reported in 39 participants (15.7%), 21 (16.3%) in the placebo group and 18 (15.3%) in the Mg-oxide group (*p* = 0.863). Other reported ADRs included abdominal pain, cramps, sleepiness, lethargy, dyspepsia, chest pain, constipation, pimples or allergic itchiness, and nausea. Likewise, there were no significant differences in ED visits (*p* = 0.798), hospital admissions (*p* = 0.765), or LOS (*p* = 0.369) (**Table 2**).

### 3.8 iMg and tMg concentrations trends across follow-up

**Figure 2:**
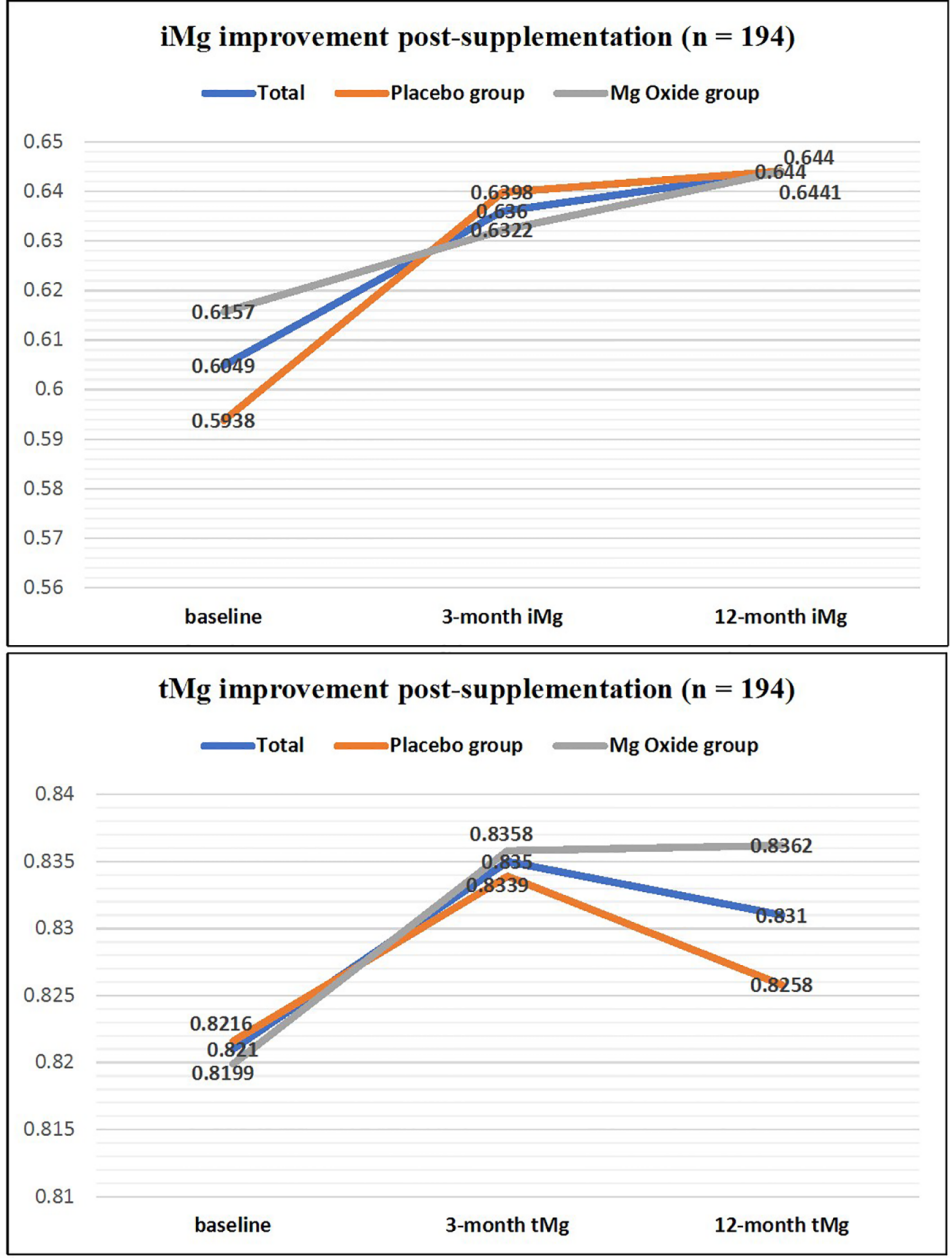
Improvement in iMg and tMg measurements post-supplementation based on PP analysis (n = 194).

As shown in **Figure 2** at baseline, the mean iMg concentration was slightly higher in the Mg-oxide group compared with placebo (0.6157 *vs.* 0.5938 mmol/L; *p* = 0.088). Longitudinal analysis showed gradual increase in iMg concentrations across follow-up visits, reaching 0.644 mmol/L at 12 months. Paired analysis showed significant within-group improvements between the 3- and 12-month visits (*p* < 0.05 for all comparisons), with greater overall improvement i in the intervention arm (*p* = 0.016). In contrast, tMg concentrations remained stable throughout the study (0.821 ± 0.082 mmol/L; *p* = 0.890) without inter-or intra-group significant changes (*p* > 0.05 across all comparisons).

### 3.9 Glycemic control trends across follow-up

**Figure 3:**
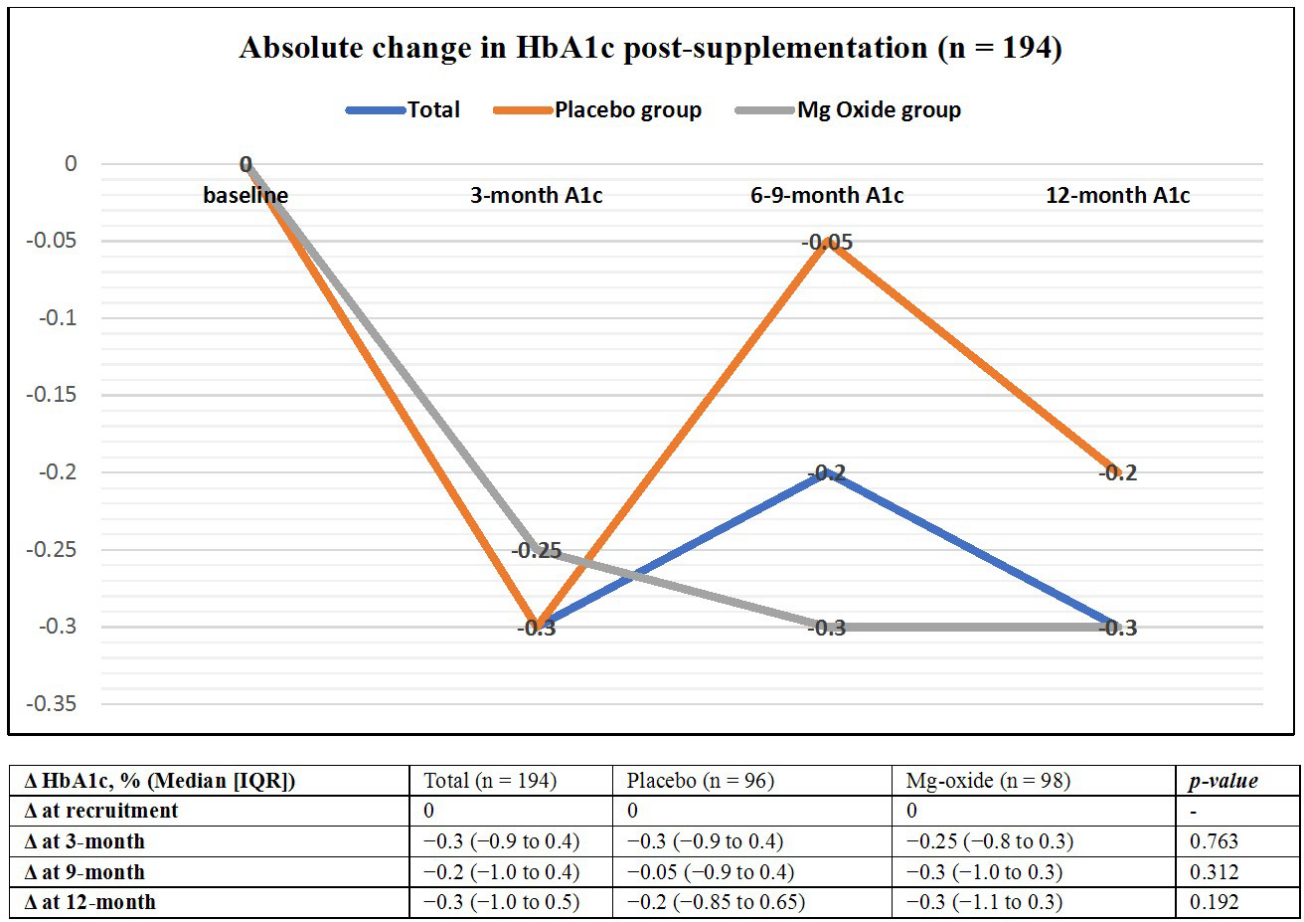
Absolute change in HbA1c post-supplementation based on PP analysis (n = 194).

Baseline HbA1c was comparable between groups (8.7% *vs*. 8.9%). Both groups showed modest declines during follow-up, reaching 8.4% in the Mg-oxide group and 8.7% in placebo group at 12 months follow-up. The absolute reductions were numerically greater with Mg-oxide (−0.3% *vs.* −0.2%) but did not differ significant across follow-up periods (*p* = 0.763 at 3 months, *p* = 0.312 at 6–9 months, and *p* = 0.192 at 12 months) (**Figure 3**).

### 3.10 Subgroup analysis

**Supplementary Table 4** summarizes the subgroup analysis of the absolute change in HbA1c following 12 months of Mg-oxide supplements. Among participants with high adherence (n = 104), median HbA1c reductions were comparable between the placebo and Mg-oxide groups (−0.4% *vs.* −0.2%, *p* = 0.877). In participants with iMg-based hypomagnesemia (n = 18), Mg-oxide was associated with a larger HbA1c reduction (−0.6% [−1.2 – −0.05]) vs.+0.25% [−0.8 – 0.8]; *p* = 0.074). A similar but less pronounced decline in HbA1c (−0.55% *vs.* + 0.3%; *p* = 0.175) was also observed in Mg-deficient individuals based on tMg (n = 20). Among participants with diabetes duration ≤15 years (n = 117), Mg-oxide produced a numerically larger HbA1c reduction (−0.4% *vs.* 0.0%; *p* = 0.0845), although this difference did not reach statistical significance.

Further, a subgroup analysis restricted to participants not using proton pump inhibitors at baseline (n = 158) showed no significant between-group differences in changes in iMg (0.01 vs. 0.01 mmol/L, *p* = 0.168), tMg (0.01 vs. 0.00 mmol/L, *p* = 0.473), or HbA1c (−0.3% vs. 0.0%, *p* = 0.201).

## 4 Discussion

This RCT provides a regionally unique contribution by evaluating long-term Mg-oxide supplementation in adults patients with T2DM using both tMg and iMg as monitoring biomarkers. It is, our knowledge, the first such RCT in Oman and among few from the Middle East (5, 18) assessing 302 mg elemental Mg-oxide over 12 months in a well-characterized diabetic cohort. Unlike most prior trials that relied solely on tMg, this study incorporated iMg, the physiologically active fraction, and leveraged a locally established iMg reference range to interpret Mg status. With 247 randomized participants, adequate statistical power, validated adherence assessment, and concordant ITT and PP findings, the trial offers robust and generalizable insights.

The primary findings of this RCT indicate a biologically reasonable effect of oral Mg-oxide supplementation on Mg status and glycemic outcomes. Participants receiving Mg-oxide showed significant increases in both iMg and tMg over 12 months, confirming effective absorption and systemic uptake of the administered formulation. The rise in iMg is especially informative, as iMg more closely reflects physiologically active, intracellular Mg than protein-bound tMg and is increasingly recognized as a more sensitive biomarker of Mg status (7, 30). These biochemical changes were accompanied by statistically significant reductions in FBG in the intervention group, whereas reductions in HbA1c did not reach statistical significance. Greater improvements in HbA1c, however, were observed among participants with baseline hypomagnesemia and those with a shorter diabetes duration, suggesting that Mg repletion may be most effective earlier in the disease course or in those with overt Mg deficiency. In contrast, secondary cardiometabolic outcomes, including BP, BMI, lipid profile, renal biomarkers, insulin dose, or clinical outcomes such as ED visits, hospital admissions, and LOS, did not differ significantly between groups. This finding suggests that a longer observation period may be necessary to detect a statistically meaningful benefit or risk. Mg-oxide demonstrated a favorable safety profile, with no signals of serious ADRs.

The observed FBG reductions corroborate prior RCTs and meta-analyses showing that Mg supplementation improves short-term glucose metabolism, with typically FBG reductions of approximately 0.5-0.7 mmol/L in individuals with diabetes, prediabetes, or insulin resistance (31–33). Such effects are most evident in patients with low baseline Mg, a pattern also observed in this trial’s hypomagnesemia subgroup (34). Several studies using inorganic Mg salts have similarly reported FBG reductions with elemental doses ranging from about 150 to 1000 mg/day over 1–4 months (34–36). Mechanistically, Mg plays a key role in nocturnal glucose homeostasis by improving insulin receptor signaling, attenuating hepatic insulin resistance, and reducing oxidative stress and pro-inflammatory cytokine activity, thereby lowering endogenous hepatic glucose output (37, 38). Mg may also modulate circadian and neuroendocrine pathways, including sleep quality, cortisol, and melatonin, which are linked to early-morning glycemia (39). Nonetheless, the FBG findings should be interpreted with caution, as FBG data were available for only approximately half of the study participants. Furthermore, measures of insulin resistance, such as the Homeostatic Model Assessment of Insulin Resistance (HOMA-IR), were not collected, limiting our ability to explore the mechanisms underlying the observed changes in glycemic control (40).

The nonsignificant, yet directionally favorable reduction in HbA1c with Mg-oxide is consistent with pooled evidence suggesting modest average HbA1c improvements (−0.16% to −0.22%) with Mg supplementation (5, 22). Prior reports have shown that higher Mg doses (~500 mg/day), longer supplementation durations (≥4 months), baseline hypomagnesemia, and shorter diabetes duration (25, 26), enhance effect size, pattern mirrored in the subgroup trends observed here. Importantly, a significantly higher proportion of participants in the Mg-oxide group achieved HbA1c <7% (14.0% *vs*. 6.0%; *p* = 0.043), supporting clinically meaningful benefit for a subset of patients (41). Longitudinal analysis showed a smoother progressive HbA1c decline in the Mg-oxide arm, versus more variable patterns in the placebo arm, consistent with behavioral and Hawthorne effects commonly reported in T2DM RCTs (42, 43). The lack of parallel Mg biomarker changes in the placebo arm supports a primarily behavior-driven rather than biologic effects. Several factors may underline the apparent discordance between FBG and HbA1c: long-standing T2DM with β-cell exhaustion, heterogeneous in baseline Mg status, the pharmacokinetics and compartmentalization of Mg, nighttime dosing favoring fasting rather than postprandial glycemia, and unmeasured confounders such as vitamin D status or dietary quality (5, 44–48).

Neutral effects on secondary cardiometabolic outcomes are broadly consistent with prior RCTs and meta-analyses (5, 49, 50). Although Mg has vasodilatory, endothelial and anti-inflammatory effects, BP reductions reported in earlier studies have generally been small and inconsistent, and more apparent in short-term trials, untreated hypertensive populations, or individuals with low baseline Mg (5, 51, 52). In this trial, participants were older, had long-standing T2DM, and were largely receiving optimized antihypertensive and lipid-lowering therapy, given the tertiary care setting, with baseline values already within near-target ranges, reducing the potential to detect additional Mg-related effects. Triglycerides, LDL-C, HDL-C, insulin dose, and BMI remained stable, aligning with previous pooled evidence suggesting that Mg supplementation exerts only minor additional effects on these endpoints in intensively treated T2DM populations (5, 18, 38) (5, 18, 53). Renal biomarkers similarly remained unchanged, supporting the renal safety of 12 months Mg-oxide in this setting and align with limited clinical evidence in T2DM (54).

From a safety perspective, this trial reinforces the favorable tolerability of chronic Mg-oxide supplementation in T2DM. Rates of ED visits and unplanned hospital admissions were low and comparable between groups, and no pattern of treatment-emergent instability was observed over 12 months. These findings are consistent with prior RCTs, systematic reviews, and large real-world cohort data indicating that Mg supplementation, including Mg-oxide, is generally well tolerated in T2DM without excess risk of acute decompensation or hospitalization (15, 55). ADRs were largely mild and gastrointestinal, dominated by diarrhea, and occurred at comparable frequencies in both groups, consistent with known osmotic effects of oral Mg salts (56). Importantly, serious ADRs, arrhythmias, and clinically relevant dysmagnesemia requiring specific intervention were rare, underscoring the acceptability of Mg-oxide as a long-term adjunct.

The findings of this trial have several important clinical implications for routine diabetes care, especially for those with low Mg status, suboptimal FBG, or shorter disease duration. Given the established association between low Mg status and poor glycemic and adverse cardiometabolic outcomes (10, 55), targeted screening and correction of Mg deficiency may represent a pragmatic strategy, a low-cost adjunct to t standard diabetes care rather than a universal intervention. Future trials should prioritize strategies that better enhance intracellular Mg^2^⁺ availability rather than relying solely on elemental intake. Approaches may include divided or daytime dosing or alternative formulations with improved bioavailability, such as sucrosomial^®^ Mg-oxide, or Mg bisglycinate, which have shown better pharmacokinetic profiles compared with conventional Mg-oxide formulations (57). Incorporating continuous glucose monitoring could detect subtle glycemic effects and nocturnal glucose patterns that HbA1c alone cannot detect. Given emerging evidence linking Mg to endothelial function and microvascular health, evaluating its impact on early neuropathic and vascular complications—especially in those with documented hypomagnesemia—also appears warranted (11). Personalized Mg dosing guided by baseline iMg concentrations and informed by population pharmacokinetic modeling represents another promising approach to refine treatment thresholds and dosing strategies.

This RCT has several limitations to consider when interpreting the findings. First, the single-center design in a tertiary care setting may limit generalizability to other clinical settings and populations. Residual confounding is possible despite rigorous randomization and blinding. Second, reliance on self-administered supplementation and behavioral outcomes such as HbA1c introduces susceptibility to adherence variability and lifestyle changes that were not fully quantified. Third, potential indirect modifiers, including vitamin D status, were not systematically assessed and may have influenced treatment response. Fourth, mechanistic endpoints such as HOMA-IR, insulin sensitivity screener, and inflammatory markers were not measured, limiting insights into other pathways underlying glycemic changes. Finally, the trial was not powered for hard clinical endpoints such as cardiovascular events or progression of microvascular complications, and the follow-up duration, while longer than many previous RCTs, may still be insufficient to detect such outcomes. Future multicenter trials stratified by baseline Mg status and key metabolic modifiers, and incorporating mechanistic biomarkers, are therefore needed.

## 5 Conclusion

This double-blind RCT provides key clinical and methodological insights into Mg supplementation in T2DM. Twelve-month Mg-oxide improved Mg status and modestly enhanced glycemic control without increasing ADRs. Although overall HbA1c reduction was not significant, more participants achieved target levels. Benefits were greater in those with hypomagnesemia, or shorter diabetes duration. Given the study limitations, Mg-oxide appears to be a safe adjunct therapy; however, larger multicenter iMg-guided trials are needed before routine guideline incorporation.

## Data Availability

The raw data supporting the conclusions of this article will be made available by the authors, without undue reservation.

## Glossary

ADRs: Adverse Drug Reactions
BMI: Body Mass Index
BP: Blood Pressure
CI: Confidence Interval
CrCl: Creatinine Clearance
DBP: Diastolic Blood Pressure
DM: Diabetes Mellitus
ED: Emergency Department
FBG: Fasting Blood Glucose
FDA: Food and Drug Administration
GDM: Gestational Diabetes Mellitus
GIP: Glucose-Dependent Insulinotropic Polypeptide
GLP-1: Glucagon-Like Peptide-1
HbA1c: Glycated Hemoglobin
HDL: High-Density Lipoprotein
HOMA-IR: Homeostatic Model Assessment of Insulin Resistance
iCa: Ionized Calcium
iMg: Ionized Magnesium
ISE: Ion Selective Electrode
ITT: Intention-to-Treat
LDL: Low-Density Lipoprotein
LOCF: Last Observation Carried Forward
LOS: Length of Stay
Mg: Magnesium
MMAS-8: Morisky Medication Adherence Scale (8-item)
OHA: Oral Hypoglycemic Agents
PP: Per-Protocol
RCT: Randomized Controlled Trial
SBP: Systolic Blood Pressure
SD: Standard Deviation
SGLT2: Sodium–Glucose Cotransporter-2
SQUH: Sultan Qaboos University Hospital
T1DM: Type 1 Diabetes Mellitus
T2DM: Type 2 Diabetes Mellitus
TG: Triglycerides
tMg: Total Magnesium.
