# Supplementary material for: The effect of oral magnesium supplementation on glycemic control and metabolic parameters in type 2 diabetes mellitus: a double-blind randomized controlled trial
Source: Front Endocrinol (Lausanne). 2026 Jul 16;17:1883483. doi: 10.3389/fendo.2026.1883483 (PMC13422152; doi:10.3389/fendo.2026.1883483)
Supplement: Supplementary file 1 [file SupplementaryFile1.docx]

**Supplementary material**

**Supplementary Table 1:** Ingredients of Mg-oxide tablets and placebo tablets.

| **Ingredients of Mg-oxide tablet** | **Content of Mg-oxide tablet** | **Ingredients of a placebo tablet** | **Content of a placebo tablet** |
| --- | --- | --- | --- |
| Microcrystalline Cellulose | 183 mg | Microcrystalline Cellulose | 680 mg |
| Polyvinyl Pyrrolidone | 65 mg | Polyvinyl Pyrrolidone | 65 mg |
| Magnesium Stearate | 10 mg | Magnesium Stearate | 10 mg |
| Silicon Dioxide | 5 mg | Silicon Dioxide | 5 mg |
| Active Mg | 300 mg | - | |

**Supplementary Table 2**. Changes in Mg status between baseline and 12-month follow-up using serum iMg and tMg concentrations

| **Mg Status** | **iMg at baseline (95% CI)** | **iMg at 12-month (95% CI)** | ***p*-value (McNemar)** | **tMg at baseline (95% CI)** | **tMg at 12-month (95% CI)** | ***p*-value (McNemar)** |
| --- | --- | --- | --- | --- | --- | --- |
| **Hypomagnesemia** | 7.29 (4.63 – 11.29) | 4.05 (2.19-7.38) | **0.011** | 8.10 (5.27-12.24) | 4.86 (2.77-8.38) | **0.046** |
| **Normomagnesemia** | 74.90 (69.09-79.94) | 68.42 (62.3373.94) |  | 89.47 (84.96-92.75) | 92.31 (88.23-95.05) |  |
| **Hypermagnesemia** | 17.81 (13.51-23.12) | 27.53 (22.30-33.46) |  | 2.43 (1.09-5.32) | 2.83 (1.35-5.84) |  |
| *iMg: ionized magnesium; tMg: total magnesium; CI: confidence interval.* | | | | | | |

**Supplementary Table 3**. Agreement between Mg measures and the correlations with HbA1c.

| **Variable Pair** | **Spearman’s ρ (*r*)**  *unless otherwise specified.* | ***p*-value** | **Lin’s Concordance Correlation Coefficient (*rho-c*)** | **95% CI** | ***p*-value**  *Bradley-Blackwood*** |
| --- | --- | --- | --- | --- | --- |
| **iMg *vs* tMg** | 0.587* | **<0.001** | 0.154 | 0.119 – 0.188 | <0.001 |
| **iMg *vs* baseline HbA1c** | −0.198 | **<0.001** |  | | |
| **iMg *vs* 12-month follow-up HbA1c** | −0.092 | 0.150 |  |  |  |
| **tMg *vs* baseline HbA1c** | −0.077 | 0.226 |  |  |  |
| **tMg *vs* 12-month follow-up HbA1c** | −0.118 | 0.064 |  |  |  |
| *iMg: ionized magnesium; tMg: total magnesium; HbA1c: glycated hemoglobin; CI: confidence interval.*  **Person’s correlation was used for normally distributed Mg measures.*  ***The null hypothesis of equal means and variances tested using the Bradley–Blackwood procedure, should not be rejected (p > 0.05).* | | | | | |

**Supplementary Table 4**: Subgroup analysis of change in glycated hemoglobin (HbA1c) post-supplementation.

| **Subgroup** | **n (%)** | **Total Δ HbA1c, % (Median [IQR])** | **Control/Placebo group** | **Intervention/ Mg-oxide group** | ***p*-value** |
| --- | --- | --- | --- | --- | --- |
| **High Adherence** | Total: 104 (100)  Placebo: 46 (44.2)  Mg-oxide: 58 (55.8%) | −0.2 (−1.0 – 0.55) | −0.4 (−0.9 – 0.5) | −0.2 (−1.0 – 0.6) | **0.877** |
| **Hypomagnesemia (by iMg) at baseline** | Total: 18 (100)  Placebo: 10 (55.6)  Mg-oxide: 8 (44.4) | −0.05 (−1.0 – 0.3) | +0.25 (−0.8 – 0.8) | −0.6 (−1.2 – −0.05) | **0.074** |
| **Hypomagnesemia (by tMg) at baseline** | Total: 20 (100)  Placebo: 8 (40.0)  Mg-Oxide: 12 (60.0) | 0.0 (−1.2 – 0.35) | +0.3 (−0.35 – 0.45) | −0.55 (−1.7 – 0.15) | **0.175** |
| **Duration of DM ≤ 15 years** | Total: 117 (100)  Placebo: 63 (53.8)  Mg-Oxide: 54 (46.2%) | −0.1 (−1.3 – 0.4) | 0.0 (−1.1 – 0.6) | −0.4 (−1.4 – 0.2) | **0.085** |
| **Not Treated with Insulin** | Total: 95 (100) Placebo: 48 (50.5)  Mg-Oxide: 47 (49.5) | −0.2 (−1.0 – 0.3) | −0.05 (−0.8 – 0.6) | −0.3 (−1.3 – 0.3) | **0.133** |
| **Not on Proton Pump Inhibitors** | Total: 95 (158) Placebo: 81(50.5)  Mg-Oxide: 77 (49.5) | -0.1 (-0.9 - 0.6) | 0.0 (-0.8 - 0.7) | -0.3 (-1.0 - 0.5) | **0.201** |
